# Assisted Living System with Adaptive Sensor’s Contribution

**DOI:** 10.3390/s20185278

**Published:** 2020-09-15

**Authors:** Magdalena Smoleń, Piotr Augustyniak

**Affiliations:** Department of Biocybernetics and Biomedical Engineering, AGH University of Science and Technology, 30 Mickiewicza Avenue, 30-059 Kraków, Poland; august@agh.edu.pl

**Keywords:** home care, monitoring, multimodal system, activities of daily living, sensors, recording

## Abstract

Multimodal sensing and data processing have become a common approach in modern assisted living systems. This is widely justified by the complementary properties of sensors based on different sensing paradigms. However, all previous proposals assume data fusion to be made based on fixed criteria. We proved that particular sensors show different performance depending on the subject’s activity and consequently present the concept of an adaptive sensor’s contribution. In the proposed prototype architecture, the sensor information is first unified and then modulated to prefer the most reliable sensors. We also take into consideration the dynamics of the subject’s behavior and propose two algorithms for the adaptation of sensors’ contribution, and discuss their advantages and limitations based on case studies.

## 1. Introduction

Nowadays, in developed countries, significant progress in the process of aging is observed—the percentage of elderly people in the population is higher than the percentage of young people. It is expected that in these countries the current 20%-proportion of people age 60 years and above will increase by 32% by the year 2050. Over 50 years between 1950 and 2000 the median age increased from 29.0 years to 37.3 years and its continued growth is estimated to be 45.5 years by the year 2050 [1].

These figures force the governments of developed countries to carry out adequate actions. They mainly consist of the monitoring of health parameters and physical activity for the purposes of prevention against all types of diseases and life risks such as falls and frailty due to the absence of systematic physical exercise, selected on the individual level. Taking care of people who need special treatment (older, with disabilities, during recovery after injuries, accidents, or serious illnesses) is not limited to satisfy their physiological or material needs, but first of all involves physical, psychological, and social stimulation [2]. As early as in ancient times, not without reason, Aristotle said that “movement is life—life is movement”. Thus, all attempts and efforts towards achieving practical support for such people by encouraging their psychomotor autonomy are of great importance.

To face the above needs, projects of technical solutions proposed worldwide aim at the non-invasive, convenient, and secure monitoring of supervised human vital signs [1,3]. Such monitoring is expected to reduce the costs of expensive medical equipment or specialized medical and rehabilitation staff and to assist non-professional individuals in taking continuous care of ill people.

Every approach to an assisted living system raises three issues:

- Adequacy of the applied sensor set;
- Intrusion of measurement devices in the subject’s environment and behavior;
- Violation of the subject’s privacy and vulnerability of the collected data.

With the rising demand for applying the new technical solutions in the field of ambient assisted living, scientific works and their outcomes are widely presented [4]. Therefore, various types of approaches of ambient sensor-based monitoring technologies detecting elderly events (activities of daily living and falls) can be found in the current literature such as non-contact sensor technologies (motion, pressure, video, object contact, and sound sensors), multicomponent technologies (combinations of ambient sensors with wearable sensors), smart technologies, and sensors in robot-based elderly care.

With the aim of non-intrusively monitoring human wellbeing at home, the domestic energy supplies can be also disaggregated in order to detect appliance usage by means of machine learning and signal processing [5]. This enables the identifying of behavioral routines, detecting anomalies in human behavior, and facilitating early intervention.

To support the independent life of seniors and people with chronic conditions and potential health-related emergencies an Internet of Things (IoT) network is implemented for continuous monitoring [6]. The solution is based on the network including mobile phones to transmit the data generated by the IoT sensors to the cloud server and the 3rd party unknown mobile relays.

Since the home environment is usually monitored by sensors collecting a vast volume of collected data, the computational methods should process it in an appropriate time [7]. This implies the need for an event-driven framework in order to detect unusual patterns in such environments.

Another important point is designing and implementing an indoor location and motion tracking system in a smart home setup [8]. The role of such a system is to track human location based on the room in which the supervised person is located at a given time and to recognize the current activity.

Since in real daily life human behavior is not so predictable, a hybrid framework for human behavior modeling could take a great role in managing the changing nature of activity and behavior. The feedback-based mechanism could be significant to recursively append new events and behavior and classify them into normal or abnormal human behavior [9].

Due to the rapid evolvement of Ambient Assisted Living (AAL), there is also the necessity of standardization, uniformities, and facilitation in the system design [10]. The paper presents the latest survey of the AAL system’s models and architectures. The authors investigated the AAL system requirements and implementation challenges, Reference Models (RM) and Reference Architectures (RA) definitions, demands, and specifications.

Simple unimodal approaches propose using a motor signal that adequately describes the state and behavior of the monitored person. This type of measurement allows not only to initiate an alarm in the dangerous or unusual situation [11,12] but also allows to specify a degree [13] and a type of daily physical activity [14]. It was also found helpful in the evaluation of rehabilitation progress and providing biofeedback to support the growth of psychological motivation and engagement in physical exercises [15].

In multimodal approaches, the activity sensors use various physical measurements and data fusion methods to provide consistent information about the subject’s activity. This usually raises a question about the adequate usage of particular sensor types accordingly to their advantages in specific scenarios. Studying numerous papers on ambient assisted living, considering personal longstanding experience, and being inspired by rules of nerve sensitivity modulation in humans, we were motivated to propose a multisensor system with an adaptive contribution of particular sensors to the final behavior classification accordingly to the present and most probable future actions. The scope of the reported research includes the analysis of the performance of the four most commonly applied assisted living sensors (three of them are wearable) in six elementary reversible activity types of the human. Based on this analysis background rules of sensor contribution have been proposed and applied to build an auto-optimizing multimodal surveillance system. The main purpose of the work is to confirm the complementary competencies of the sensors and benefits resulting from their adaptive contribution in realistic assisted-living scenarios.

Consequently, the main novelty presented in this paper is the concept of a system for the recognition of human daily activity that adapts the process of multimodal data fusion following the criteria of sensitive, selective, non-intrusive, and privacy-protective measurements (Section 3).

To this point, we tested basic behavioral measurements with a custom-built multimodal surveillance system (Section 4), registered and interpreted many different vital signs from supervised people with low-cost and easy-to-use sensors, and compare their sensitivity and selectivity of action recognition. Elements of this system have been developed as the result of different previous projects focused on single sensing modalities such as control of the living environment with the eye movements [16], motor cortex rhythm [17], facial information [18], and sound recognition [19]. The cooperation of several sensors with different characteristics has been proposed in two other projects dedicated to the supervision of humans during sleep [20,21]. We also contributed to the research aimed at the development of sensor networks for supervising the human in motion based on motion patterns from wall-mounted cameras [12,22] or data from wearable devices [23,24]. Finally, two approaches of sensor data fusion from multimodal sensing systems have been proposed in [25,26].

This research summarized in Section 5 paved a way to propose two algorithms for continuous modulation of the extent of influence from each particular sensor to the final recognition (Section 6). Section 7 presents the case studies, Section 8 contains discussion and Section 9—concluding remarks.

## 2. State-of-the-Art

Multimodal systems used in home monitoring of people can be considered in the context of simultaneous acquisition of either a variety of human biomedical signals—motion, EEG, ECG, acoustic [20,21,23,24,25], or the same signal (motor) but using different sensing methods. In this paper, we focus on the motor activity of the subject and propose a multimodal motion recording system. Consequently, the review below includes basic approaches to human motion sensing.

### 2.1. Behavior Sensing Techniques

Scientific work of Suh and Park [27] presented a monitoring system based on motion sensors of various types: inertial (built of three-axis gyroscope and three-axis accelerometer, attached on a foot back) and pressure (FlexForce A201 from Tekscan company, positioned under a heel). Eight ADL states were analyzed: sitting, walking, walking up and down (walking on an uphill and downhill road), running, running up, running down, and standing. For the estimation of the above states, the filter of the Hidden Markov Model (HMM) was proposed. The 802.15.4 wireless modules were used to identify where the activities were taking place. During detection, such activities as walking or running, the measurements were performed using inertial navigation algorithms.

In [28], surface electromyography was compared with accelerometry in the detection of eleven Functional Motor Activities (FMAs). The sensors were placed on the limbs and trunk. The features vectors, extracted in the signal processing, were used as input data to the Multilayer Feedforward Neural Network (MFNN) with two hidden layers (of 44 and 22 neurons). With a classification error of 10% for both types of sensor, the sensitivity was over 80%, and the specificity was over 97%. The sensitivity for signals received from the ACC was almost 5% higher than from the EMG. Further analysis showed that for some activities the classification based on the EMG sensor is much more sensitive. Across persons, the ACC signal was characterized by less diversity than the EMG. These results were the motivation for the authors to carry out preliminary tests with a hybrid system, which consisted of five ACC and three EMG sensors. With a classification error below 10% the used combination of sensors brought slightly better results than those given separately by the eight-element set of ACC and EMG.

Hsieh et al. [29] designed and built an independent system—an exoskeleton for the monitoring and analysis of the particular events and phases of gait. In order to measure the plantar forces’ distribution and angles of the hip and knee joints four force sensors FlexiForce (under the first metatarsal head, forth metatarsal head, hallux, and heel) and two angles changes sensors—potentiometers (in the hip and knee joints) were used. The results obtained from the proposed system and the reference systems (Vicon and dynamometer platform) were similar.

Mizuno et al. [30] introduced a multimodal system for the recognition of ADL activities of monitored persons. The system integrates piezoresistive pressure sensors, a motion detector placed in a watch, a sound sensor in glasses, an ultrasonic sensor (closed in a pen) measuring a distance from a ceiling, and a position sensor (Bluetooth and GPS). The proposed system enables the detection of walking, running, standing, eating, talking, and office work.

In [31], the physical activity of persons during rehabilitation after the stroke was monitored. For this purpose, the integrated three-axis accelerometric and one-axis gyroscopic sensors (positioned bilaterally to the subjects’ ankles and wrists) were used. Two accelerometers and a pressure sensor were attached to the cane used by the examined person. Measurement data were recorded during level walking, walking carrying an object, walking on an uneven surface, walking up a ramp, walking down a ramp, walking up a flight of stairs, walking down a flight of stairs, walking over an object, pivoting, and opening a door. Each motor activity was identified by a neural network. For all activities at an average specificity of 95%, the sensitivity ranged from 75.1% to 97.4%. Then, the use of the cane was studied in the context of a particular type of activity. The studies were performed based on the measurements data from sensors located on the cane. 

An extensive review of methods used in ambient assisted living systems was provided in [32].

The variety of methods used for sensing particular behavior patterns (e.g., fall detectors) raises the question of their substitution or complementary use. This issue was studied in our group [33] and several other authors provided comparative results for the efficiency and accuracy of different sensor types in specific everyday living events. These findings paved the way to a concept of multimodal sensing where sensors of different types are used in the following scenarios:

- Simultaneous: information from both sensors are gathered concurrently and fused together to yield features of higher sensitivity and specificity;
- Complementary: information from sensors is switched selecting the best sensor accordingly to the changes in recording conditions (e.g., indoor/outdoor).

While the simultaneous scenario has been applied in numerous proposals, the complementary scenario is also worth studying in a pursuit for continuous surveillance of a mobile human. Consequently, surveillance of physiological parameters may be employed in the healthy population as an essential part of prevention programs and on the other hand, ill or disabled people will not be sent to their beds or premises without the chance of physical exercise or a social life. 

An alternative concept was proposed in [34]. Five sensors: pulse, chest accelerometer, limb accelerometers, camera, and microphone were used in pairs for the detection of seven elementary poses, which in turn contributed to the representation of actual behavior. In that previous work, we used graph representation with node values standing for pose contribution and edge flow representing the activity in time. This approach used complementary premise-fixed and wearable sensors, simple yet reliable algorithms for recognition of elementary poses, and a concise representation of any behavior, even unknown at the setup stage.

### 2.2. Data Fusion Techniques

One of the most cited is the work by Boonma and Suzuki [35], which presents the basics of biologically-inspired architecture for Sensor Networks (BiSNET) with implemented key biological mechanisms such as energy exchange, pheromone emission, replication, and migration. The authors evaluate the BiSNET for oil spill detection in the coastal environment. The network is based on agents without a centralized service to coordinate them, thus it is lightweight, scalable, and self-healing. This means the sensor nodes autonomously adapt their states and data transmission according to dynamic changes of conditions, retain their power efficiency, against the increase of network size (up to 600 nodes), and collectively detect and eliminate false-positive data. Cohen and Edanb [36] propose a sensor fusion framework that adaptively selects the most reliable sensor set and the most suitable algorithm. To this point, the algorithm implements measures continuously quantifying sensor performance. The concept has been software simulated with a grid-map paradigm, logical sensors, and performance measures to allow the random setup of sensors producing multiple data types. The performance was measured as a difference of each particular setup and the final fused map, which has to be known beforehand. The sensor re-configuration procedure is applied once a low-performing sensor is detected.

The system presented by Marti et al. [37] is built with several sensors and a centralized automatic reasoning module that integrates partial descriptions with contextual information of the system, and combines available sensor data, to produce a fused output that best satisfies the goals following given ontology. The system is robust to temporary sensor unavailability, variable reliability of sensor information, and supports on-the-fly redefining its goals. The proposal has been implemented and tested in the ground vehicle navigation. 

A comprehensive review of the state-of-the-art techniques on multi-sensor fusion in the area of BSN can be found in [38]. The paper particularly focuses on physical activity recognition and widely discusses the data fusion pros and cons at levels of data (suitable for homogenous sensor set), features, and decisions (allowing for the combination of data from heterogeneous sensors). Moreover, centralized, distributed, and hybrid approaches to collective decision making are studied. Although a waste literature review is presented, only one example of context-adaptive fusion was provided in the work by Cook et al. [39]. 

Koping, Shirahamaand, and Grzegorzek [40] address the need for a general data fusion framework for a specific smartphone-based multi-sensor body area network. Since the framework is dedicated to a general-purpose surveillance system, it supports the heterogeneous sensor set and the data fusion is performed on the feature vector level through code-based learning. Specific signals are first processed at the sensors with adequate feature extracting algorithms. This approach is also used in the proposed solution; however, we do not follow the static data fusion paradigm. 

Very recently Lin et al. [41] proposed a smart sensors data fusion system targeted to support stable, safe, and efficient medical patient-robot interaction. The medical services provided by autonomous robots require real-time monitoring of the state of both users. To this point, various sensor, communication, robot, and data processing technologies have been applied. The proposed hybrid body sensor network architecture is based on multi-sensor fusion employing an interpretable neural network. However, the data integration process seems to be fixed for the given patient. Bazo et al. [42] propose the combination of radiofrequency-based positioning and computer vision-based human pose estimation as a tool for behavioral analysis and activity recognition. The two subsystems have complementary properties i.e., the radiofrequency localizer solves the occlusions that may occur in the computer vision detector, and the computer vision subsystem increases the accuracy of positions measured with the radiofrequency localizer. This model falls in the larger category of bimodal position and activity sensing systems also developed by other authors for analysis of shoppers [43,44], pedestrians [45,46], or just human pose recognition [47]. Both subsystems are independent and separately process the RF and RGBD sensors produced data. The sensor fusion module uses the tag and skeleton and iteratively seeks for its stable state expressed by maximizing data persistence. The priority of visual or radiofrequency data is used solely to avoid ghosting. 

He et al. [48] give a critical review of state-of-art solutions for scalable fault-tolerant information fusion in a distributed wireless sensor network. The authors indicate the most challenging areas in sensors application, which are different sensing modalities enriching the robustness but demanding more than simple fusion of homogenous data and a wide range of uncertainties in sensing and communication (misdetection, false alarms, unavailability, or delays). The paper also highlights several interesting areas of future improvements such as mutual calibration and verification of data consistency. 

Proper instrumentation and interpretation software enable detecting particular events and classifying human behavior in several categories of risk. Extending this scope leads to a continuous predict-and-verify scenario, where the detection of unexpected behavior provides signs of possible health setbacks [49]. In that previous research, the information of the currently identified pose was not utilized to improve the sensing performance of the current state nor prepare the sensing system for the most probable subject pose. A novel concept stemming from our previous studies is presented in this paper. It combines behavior prediction and sensor reconfigurability schemes into a behavior tracking system that continuously adapts the sensor contribution to the present and most probable future activity of the supervised subject.

## 3. Concept of Adaptive Sensing

The concept of continuous adaptation of sensors’ contribution in a multimodal system originates from rules of information propagation in living neural systems. Let us shortly recall two different types of chemical synapses: ionotropic, with a quick and short synaptic response, specialized in fast sensory or executory, excitatory or inhibitory pulse messaging, and metabotropic, with a delayed and long-standing response, being primarily responsible for the modulation of pulse conduction. All mammals select the dominating and auxiliary senses that they actually use to perceive the surroundings thanks to these two complementary types of synaptic junctions. 

Mimicking the above-mentioned natural rule of neural modulation in a technical multisensor assisted living environment requires solving two issues:

- Determining competence areas and performance hierarchy in a given sensor set;
- Specifying data stream modulation rules, allowing to adapt each sensor’s contribution to a final decision.

Initially, we assume each sensor to have an exclusive sector of competence area, where no other sensor is applicable, and its complementary sector, where it competes with one or more other sensors. Although the accuracy and reliability are most naturally selected as competence criteria, a variety of other parameters are applicable in a real surveillance system: availability, intrusiveness, energy consumption, etc. Moreover, the cooperation of two sensors in a common competence sector yields valuable information about the coherence of their data streams, which may be useful in other scenarios to assess the quality of measurements relying only on the auxiliary sensor (e.g., when the principal sensor data are unavailable).

In the following sections, we develop this concept by examining the sensor set and sensor-specific preprocessing software (Section 4) in an experimental detection of human motor activities (Section 5). The discussion of the experiment outcome is followed by a proposal of two data stream adaptation algorithms (Section 6) and the presentation of a use case (Section 7). The discussion and future remarks (Section 8) conclude the paper.

## 4. Experimental Examination of the Sensor Set

### 4.1. Components of the Sensor Set

**Figure 1:**
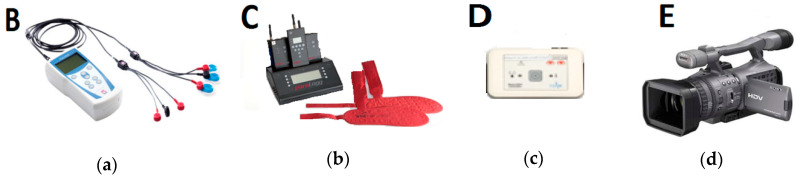
Motion signals measurement devices: (**a**) sensor B—a wireless (WLAN) EMG biopotentials amplifier ME6000 (Mega Electronics) with MegaWin software; (**b**) sensor C—a wireless foot pressure measurement system ParoLogg with Parologg software; (**c**) sensor D—ACC Revitus module with a dedicated software; (**d**) sensor E—a digital video camera Sony HDR-FX7E.

All experiments were carried out indoor in a large room (approx. 150 m^2^) by means of four different motion signals measurement devices (Figure 1): a wireless (WLAN) EMG biopotentials amplifier ME6000 (Mega Electronics) with MegaWin software (B), a wireless feet pressure measurement system ParoLogg with Parologg software (C), ACC Revitus module with dedicated software (D), and a digital video camera Sony HDR-FX7E (E) [50]. Table 1 illustrates a sampling frequency for each of the used sensors:

Eight-channel electromyographic signals were surface recorded from the muscles of both lower limbs: quadriceps—vastus lateralis (1), biceps femoris (2), tibialis anterior (3), gastrocnemius—medial head (4). Time-series foot pressure signals were obtained from the 64 built-in pressure sensors insoles (each foot insole has 32 independent sensors). A three-dimensional accelerometric signal was recorded with the use of Revitus located on the human sternum, while for video measurements a digital camera placed on the left side of the examined person was set up (720 × 576 pixels).

### 4.2. Preprocessing of the Measurement Data

**Figure 2:**
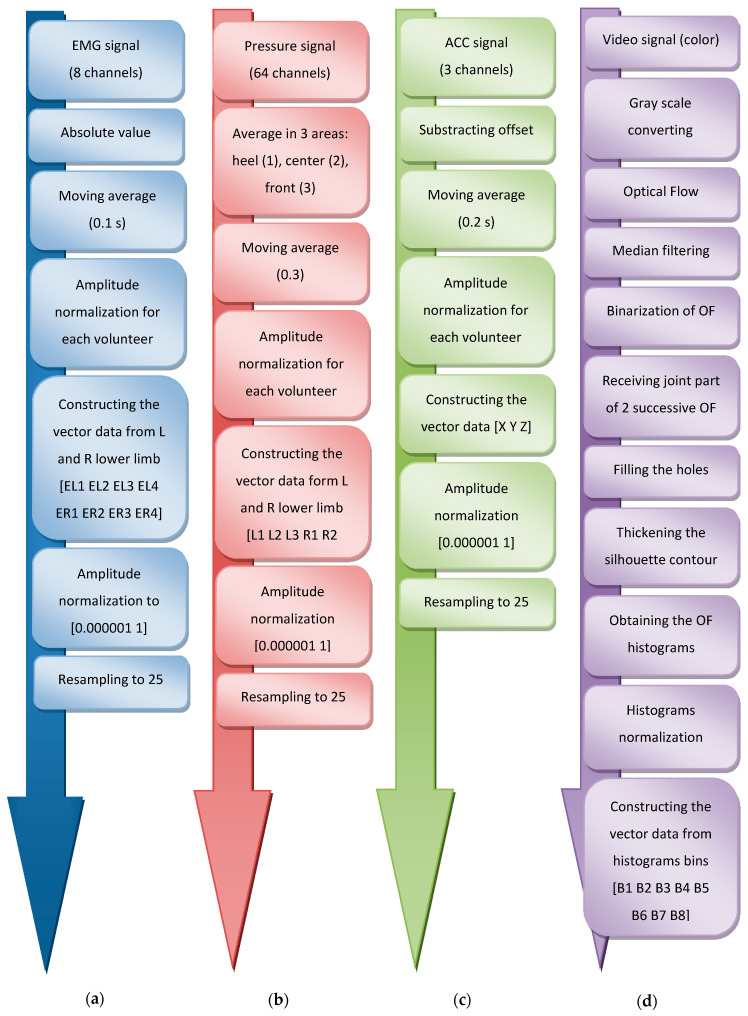
Steps of processing the measurement data from sensors: (**a**) B; (**b**) C; (**c**) D; (**d**) E. L—left, R—right.

The successive steps of processing the measurement data from each of the sensors B ÷ E were presented and described in detail in [50]. The scheme in Figure 2 illustrates the main parts of the proposed signals processing. 

The sensors were used individually and in sets of two to four sensors. The classification of motor activities was based on feature vectors recorded by one to four sensors simultaneously. The feature vectors for each setup are presented in Table 2. In the case of multiple sensors, we simply combined the feature vectors of each sensor.

### 4.3. Materials

**Figure 3:**
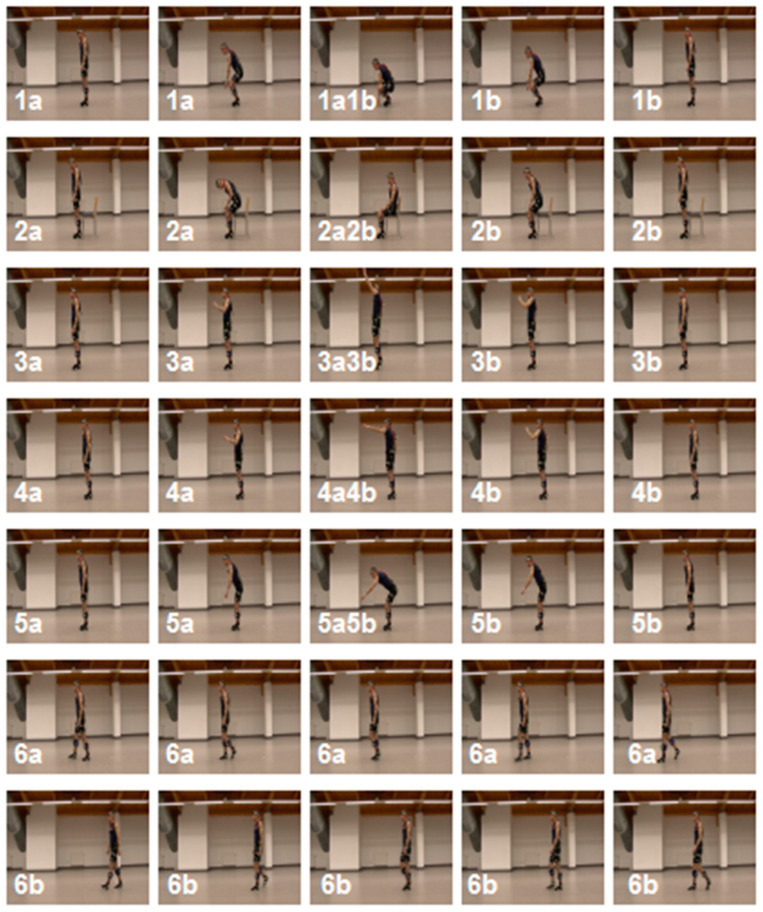
Selected (first, middle, and end, respectively) video frames presented the investigated movements (activities 1a ÷ 6b). Transitions between the specific successive activities are named 1a1b ÷ 5a5b.

In the experiment, 20 volunteers performed 12 selected physical activities (1a ÷ 6b, Figure 3) with about 30 repetitions (19 ÷ 46) for each one:

- Squat (1a) and getting up (1b) from a stand position;
- Sitting on a chair (2a) and getting up from a chair (2b) to a stand position;
- Reaching (3a) and return from reaching (3b) the upper limb forward in the sagittal plane (standing);
- Reaching (4a) and return from reaching (4b) the upper limb upwards in the sagittal plane (standing);
- Bending (5a) and straightening the trunk (5b) from bend forward from a stand pose in the sagittal plane;
- A single step with the right (6a) and the left (6b) lower limb (stance phase).

### 4.4. Feature Classification Methods

Supervised classification of the selected motor activities was performed with the use of *k*-NN (*k*-Nearest Neighbors) method and Manhattan metric. The sizes of learning and test sets were in the ratio of 1:3. With the final results presentation in mind, several variables were introduced [50]:

- Correctness of recognition for all volunteers—*R_s a_*;
- Calculation error of *R_s_a_* − *U_s_a_*—a measure of the results dispersion comes from inter-subject differences (weighted standard deviation due to different numbers of activity repetitions for each volunteer);
- Percentage of correct recognitions for all activities and all volunteers—*R_s_ALL_*;
- Calculation error of *R_s_ALL_* − *U_s_ALL_*;
- Percent recognition for all activities—*R_s_V_*;
- Calculation error of *R_s_V_* − *U_s_V_*—a measure of results value dispersion arising from differences between different activities (weighted standard deviation due to different number of repetitions of each activity for each volunteer);
- Calculation error of *R_s_V_* − *U_s_ALL_*—a measure of the dispersion of the results due to recognitions of the individual activities.

## 5. Sensor Set Performance Results

**Figure 4:**
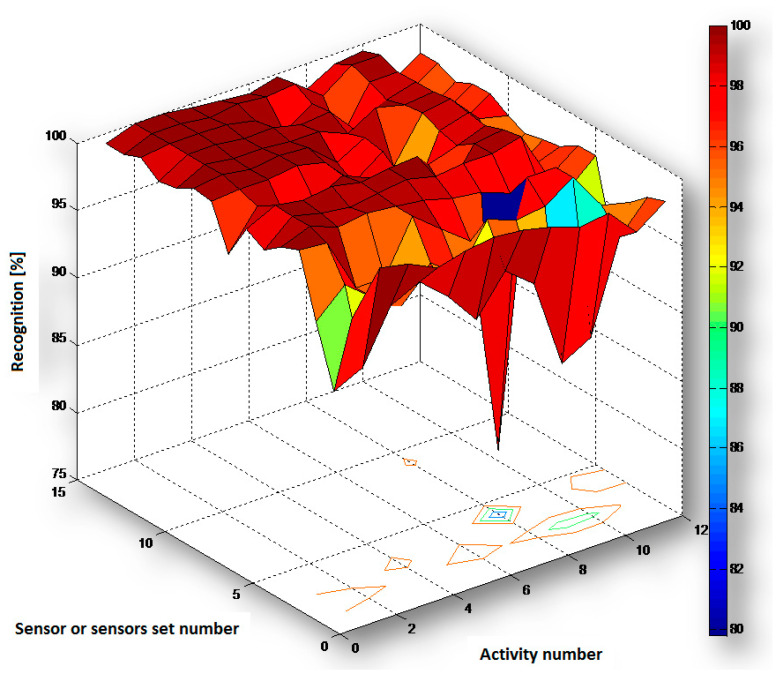
Chart of recognition *R_s_a_* (in %) for activities 1a ÷ 6b (numbers 1 ÷ 12) and for sensors B ÷ E and their sets (numbers 1 ÷ 15).

**Figure 5:**
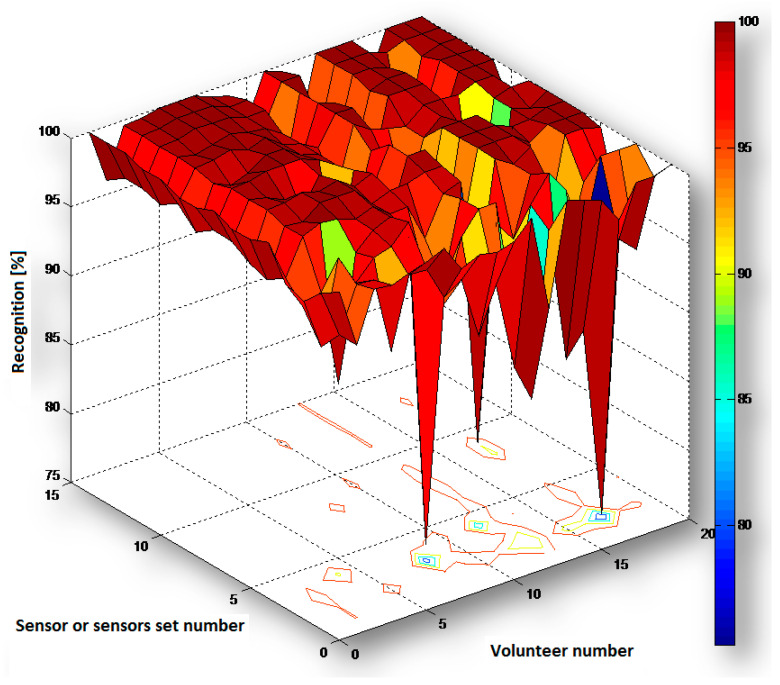
Chart of recognition *R_s_V_* (in %) for volunteers V1 ÷ V20 (numbers 1 ÷ 20) and for sensors B ÷ E and their sets (numbers 1 ÷ 15).

Based on the data presented in Table 3 and Table 4 and Figure 4 and Figure 5 we concluded that the measurements carried out simultaneously with two, three, or four sensors lead to a significant improvement of recognition reliability.

Matrices of the recognition errors (in %) of the individual motor activities 1a ÷ 6b in the test set for all people together for sets of sensors B ÷ E (BC, BD, BE, CD, CE, DE, BCD, BCE, CDE, BDE, BCDE) are shown in Table A1, Table A2, Table A3, Table A4, Table A5, Table A6, Table A7, Table A8, Table A9, Table A10 and Table A11.

The experiment results prove that the overall activity recognition performance (right columns of Table 3 and Table 4) can be improved by adapting the sensor set and the features used to the particular action and to the particular subject. This statement is a background of the proposed adaptation algorithms presented in Section 6.

## 6. Reliability-Driven Sensor Data Fusion

### 6.1. General Assumptions and System Design

**Figure 6:**
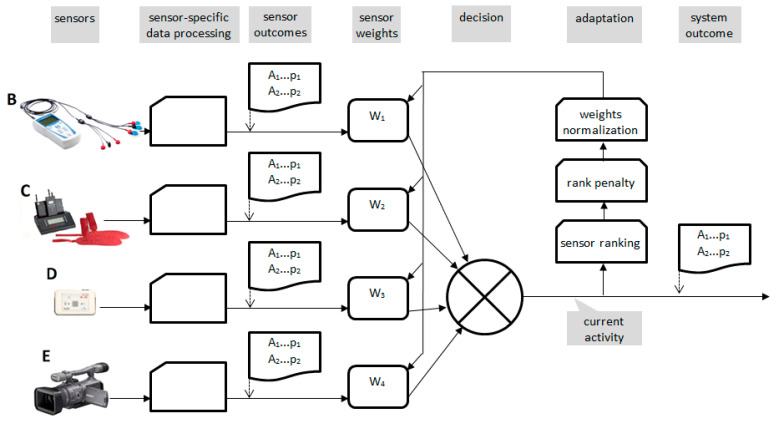
Flow of sensors’ data modulator adapting a multimodal assisted living system to presently detected behavior.

**Figure 7:**
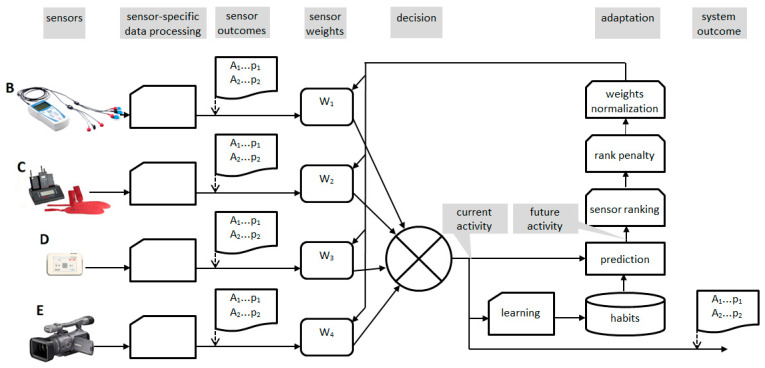
Flow of sensors’ data modulator adapting a multimodal assisted living system to most probable future behavior.

The general architecture of a multisensory environment for assisted living consists of sensors, dedicated feature extraction methods, and modality selectors. The proposed innovation replaces the selector by a modulator using weight coefficients *W_k_* (Figure 6) to prefer the most pertinent features while discriminating the others. As the sensors use specific signals (muscular, pressure, acceleration, and video), one of the consequences of replacement of the feature selector by a modulator is the necessity of uniform representation of all features. To this point, the feature calculation step uniforms the information update rate and normalizes the feature values. The output of each sensor is given as a probability-ordered list of activities {*A_i_*, *p_i_*} (see Figure 6 and Figure 7).

Three coefficients are proposed to modulate the influence of each sensor on the final decision about the detected activity. These are listed and shortly explained below.

*H_k_* is an activity-independent coefficient characterizing each sensor cost including hardware, installation, and maintenance as well as human factors like acceptance of each particular sensor set (cameras at home, accelerometer belt or bands, electrodes, etc.); all these factors we consider to be constant in time thus these values need to be evaluated once per subject. In order to efficiently adapt the sensors’ choice, extreme values of H_s_ should be avoided. 

*R_k_*(*A*) is an activity-dependent factor of reliability; as it was demonstrated in Section 5, sensors show different performance in the detection of basic daily activities of the human; accordingly, in the system paradigm, *R_s_* is the primary factor adapting the contribution from sensors to the current activity of the monitored subject.

*L*(*n*) is a penalty factor that discriminates the influence from sensors depending on their position n on the reliability ranking in determining the activity *A* by sensor *k*; the actual penalty factor is calculated based on a coefficient *p*: low values of *p* equalizes the ranking list what makes the system mostly working with multiple sensors and avoiding the worst, while a high value of *p* prefers the winner to be a unique working sensor:

(1) $$L\left(n\right)=n^{-p}\;n\in \left\{1\ldots 4\right\},\;p\in \left(0.1\ldots 10\right)$$

The contribution of each sensor *k* may be thus determined as:

(2) $$C_{k}=H_{k}\cdot R_{k}\left(A\right)\cdot L\left(n_{k,\;A}\right)$$

and normalized over the whole set of sensor weighting coefficients:

(3) $$W_{k}=\;\frac{C_{k}}{\sum_{n}C_{n}}$$

Accordingly, with the currently detected subject’s action, the system automatically adapts the feature set (Table 2) to optimally detect the present action. The optimization criteria may be freely selected from variables presented in Section 4.4 and used jointly with other attributes (including non-technical such as acceptance, usage cost, etc.). To keep the presentation simple, we use the correctness of recognition (given in Table 3). In a real system, besides the subject action, the selection of sensors also takes into account constant factors like costs and availability or acceptance of a sensor by individual subjects.

Instead of applying recognition correctness generalized for all volunteers, an individual table, equivalent to Table 3 may be built for each supervised subject. The personalization of the multisensor environment improves the individual performance (compare columns in Table 4) but requires a set of exercises performed under the supervision of a human assistant who annotates the activities and checks the recognition correctness (or other optimization criteria).

Based on selected optimization criterion (in our example: generalized correctness of recognition, Table 3) a hierarchy of feature vectors is built for each detected activity. Taking the action “bending” (5a) as an example, we have sensor set hierarchy:
(highest)BDE;
(BD, BE, DE, CDE);
(BCD, BCDE);
(CE, BCE);
CD;(lowest)BC.

It is noteworthy that BD yields better results than BCD, therefore the use of more sensors does not lead to better results, and adding a sensor (C in this case) may degrade the recognition correctness.

The modulation of the sensor’s contribution presented above is confirmative. Firstly, the detection is roughly made with a possibly not optimal sensor set and then confirmed with an adapted set. The modification closes the information loop and, like all kinds of feedback, raises the stability issue if the action detected with adapted features does not match those initially detected. The other drawback of confirmative detection is related to possible erroneous first detection leading to an even less optimal sensor set and confirming the erroneous decision.

### 6.2. Stability Condition for Modulated Sensor Set

The stability issue in a sensor set with modulated contribution can be solved by limitation of the weight modulation range. Let *f* be a function *A* = *f*(*S_k_*;*W_k_*) assigning a unique subject’s action *A* to specific sensor outputs *S_k_* modulated by *W_k_*. This means all probability values *p_i_* of given activities *A_i_* from sensor *k* are multiplied by *W_k_*:

(4) $$A=f\left(S_{k};\;W_{k}\right)=\;\underset{i}{\max}\sum_{k}\left(\left\{A_{i};p_{i,k}\cdot W_{k}\right\}\right)$$

Let *m* be a function *W_k_* = *m*(*A*) modulating the contributions from sensors *S_k_* to maximize the reliability of the recognition of *A*. Therefore, the modulator is stable if:

(5) $$\forall f:f\left(S_{k},W_{k}\right)=f\left(S_{k},m\left(A\right)\right)$$

which means the modulation does not influence the current recognition result. 

Since we cannot expect the recognition result to be a linear function of the modulation depth, we propose an iterative try and fail algorithm finding the modulation limits. To find the value of *W_k_*, between the original *W_k_*_1_ and the desired target *W_k_*_2_ the algorithm repeatedly bisects an interval and then selects a subinterval in which both ends yield different actions for further processing.

(6) $$W_{k}=W_{k1}\;\mathrm{when}\;f\left(S_{k},\;W_{k}\right)=f\left(S_{k},\;W_{k1}\right)$$

(7) $$W_{k}=W_{k2}\;\mathrm{when}\;f\left(S_{k},\;W_{k}\right)=f\left(S_{k},\;W_{k2}\right)$$

All necessary steps of the modulation algorithm are performed within the subject state sampling interval. New data gathered from the sensors are processed with optimized sensors’ contribution and confirm the detected subject’s action. 

The stability issue can be also avoided by applying a sensor set consistency rule. This rule uses the past sensor set as a reference and requires the new set to be as similar as possible. Continuing the example given in Section 6.1 if “bending” has been detected with BE sensors and a “straightening the trunk” (5b) occurs thereafter, the sensor set hierarchy is the following: 

(BD, BDE);

(BE, DE);

(CE, BCD, BCE, CDE, BCDE);

CD; 

BC. 

Maintaining the BE configuration is preferred over changing to DE, despite their equal performance, for the stability reason.

### 6.3. Predictive Modulation of Sensors’ Contribution

One may question the purpose of optimization if it only confirms the result of recognition already made. Fortunately, in most assisted living environments, the prevention of dangerous events is stressed as a primary goal, their architecture usually includes an artificial intelligence-based system for learning of the subject’s habits and detecting unusual behavior as a potential sign of danger. Such systems gather the information of individual habits in a form of database learned and updated from real past behavior records. Such a database provides activity statistics, but, more interestingly, for each given activity the most probable next activity can be determined. We propose to use the information from the individual’s habits database to predict the subject’s upcoming action and adjust the sensor’s contribution accordingly (Figure 7). The modulation is still made accordingly to the stability requirements (see Section 6.2), but the sensor’s contribution now adapts to the most probable next subject’s action. 

Introducing the habits database in the feedback path has two benefits:

- Prediction of upcoming action takes into account multimodal time series instead of single points, what stabilizes the prediction in case of singular recognition error;
- Focusing on optimal recognition for current action makes the system conservative (i.e., expecting a stable status), whereas optimizing for future action makes it progressive (i.e., awaiting changes of the status).

## 7. Case Studies

### 7.1. A Compound Action

The proposed sensor’s contribution modulation technique was analyzed in a previously proposed multisensor environment for assisted living [33]. We also used previously recorded data from 20 volunteers (8 women and 12 men, aged between 22 and 61 years), acting accordingly to predefined realistic scenarios. Table 5 presents an example compound action of searching a book on a wall-mounted shell, consisting of elementary poses (defined in Section 4): squatting (1a, 1b), reaching forward (3a, 3b), reaching upward (4a, 4b), and bending (5a, 5b).

Multiple repetitions of patterns in the habits learning phase and opposed direction of elementary poses labeled with a and b facilitate correct prediction of subsequent poses and respective adaptation of sensors’ contribution. In the studied case, no abrupt corrections in the sensor set were necessary, consequently, changes of weighting coefficients were linear and not restricted by stability limits. The smoothing influence of prediction on sensors’ modulation is also revealed in Table 5. Nevertheless, studies of correct work of the system for unexpected activities and possible errors in stabilizing the algorithm need a recording of human performance according to purposely designed misbehavior.

### 7.2. Change of Environment

In this scenario, we assume that a walking subject (alternating activities 6a and 6b) goes outdoor and sensor E (the video system) no longer provides reliable data. Since the sensor set hierarchies (Table 3) are the following:

- For 6a:
  DE;
  (BCE, BCD, BDE, BCDE);
  (BD, BE);
  BC;
  CDE;
  (CD, CE).
- For 6b:
  (BC, BD, BCD, BCE, BDE, BCDE);
  CDE; (BE, DE);
  CD;
  CE.

The most reliable sets common for both activities are BCE, BCD, and BDE, and after the elimination of sensor E data, the recognition relying on sensor D (accelerometer) data has equivalent correctness. However, if the subject changes the activity, the equivalence of data from E and D is no longer guaranteed (see 1a in Table 3 as an example). For this reason, sensor B starts to be taken into consideration and the system prefers using BD.

In the case of an opposite event (i.e., the subject enters indoor), switching back to video-based sensors is not justified by the possible improvement of recognition correctness, but the video sensor will be more comfortable for the subject than the first choice accelerometer due to having one sensor less to wear. In case the subject decides to take off the accelerometer belt, the persistent consistency of information from B and E will cause a fast return to sensors BE instead of DE. 

### 7.3. Cooperation of Sensors

The cases presented in 7.1 and 7.2 assume the presence or absence of a sensor and do not turn to account the full potential offered by modulation of contributions from multiple sensors to the activity recognition. Here we assume that: (1) all sensors are available but attributed by a quantitative variable of cost and (2) the subject performs a compound action. The modulation is then expected to continuously calculate and maximize the correctness-to-cost ratio. To this point, data on recognition correctness given in Table 3 are considered to be discrete samples in a continuous space of possible actions. The system is then expected to detect actual behavior as composed of simultaneously occurring elementary poses (see [34]) and pose contribution are taken into account to select the best sensor set. Adopting the data from the experiment (Table 3), we assume the subject is simultaneously getting up from a chair (2b) to a stand position, and reaching the upper limb forward in the sagittal plane (3a). In the first part of the action, 2b dominates, in the middle part the contribution, 3a takes over and dominates in the terminal part (e.g., reaching a book on the shelf). To show the modulation process we assume that we only have BCD sensors (no video sensor) and only two of them available at a time. Accordingly to the data in Table 3, we have hierarchies:

- For 2b:
  BC;
  BD;
  CD...
- For 3a:
  BD;
  (BC, CD)...

Since the CD sensor pair is the least favorable, we are going to use sensor B (EMG signal) and, in the course of action, modulate the contribution from C (pressure) and D (acceleration). Sensor C is then successively replaced by D and BC becomes BD as the action initially resembling 2b is more and more similar to 3a.

### 7.4. Unexpected Change of Action

The last presented case assumes that the subject stands up from the chair (2b), reaches forward (3a) and, instead of returning from reaching (3b) which was the most probable action, bends (5a) searching for the book on a lower shelf, then instead of return from bending (5b) he or she directly sits back to the chair (2a). Therefore:

- In action 2b, the sensor priority set is:
  BC;
  BD;
  CD...
- In action 3a, the sensor priority set is:
  BD;
  (BC, CD)...
- In foreseen, but not actually performed action 3b, the sensor priority set is:
  (BE, BD, BDE);
  BCD;
  (BCE, BCDE);
  BC...
- In action 5a performed instead, the sensor priority set is:
  BDE;
  (BD, BE, DE, CDE);
  (BCE, BCDE)...
- In foreseen, but not actually performed action 5b, the sensor priority set is:
  (BD, BDE);
  (BE, DE);
  (CE, BCD, BCE, CDE, BCDE)...
- In action 2a the sensor priority set is:
  (BCE, CDE, BDE, BCDE);
  (BE, CE, DE, BCD);
  BD;
  BC;
  CD...

**Figure 8:**
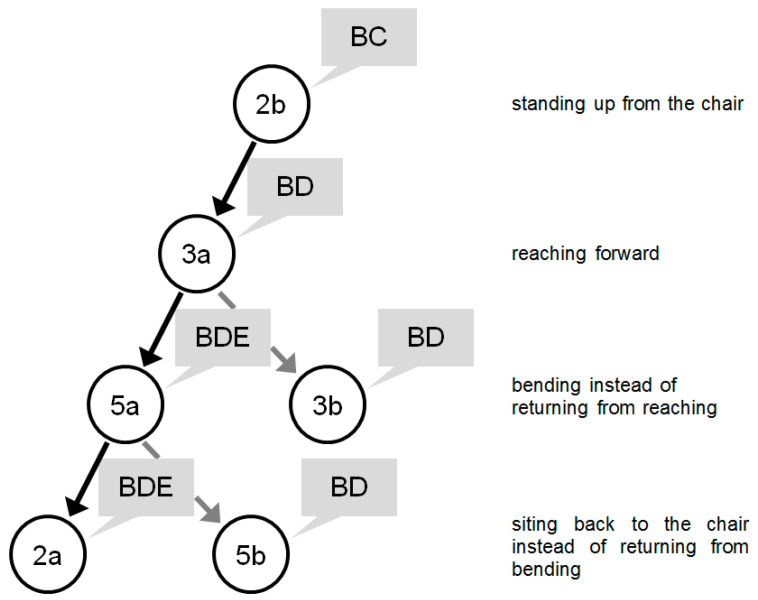
Activities performed in example 7.4 (activities are listed in circles and sensors in grayed rectangles) with unexpected activities and system decisions: at (5a) sensor E is included additionally and at (2a) the usage of sensor E is maintained.

The process of sensor selection may be in this case presented in a tree as in Figure 8.

## 8. Discussion

The results showed that it is possible to recognize the selected motor activities of everyday life with high reliability by using a different kind of individual sensor as well as their 2-, 3-, or 4-elements sets. Although some activities are recognized with less reliability with the use of some sensors, in such case there is a possibility to successfully use the data from other sensors (see discussion and conclusions in [50]) or sensors sets for which the outcome is more reliable. As can be observed from Table 3 and Figure 4 the recognition with the use of sensors sets very often has higher values (94.1–100%) than with the use of the individual sensors, for any type of activity. The same observation can be also taken from Table 4a, Table 4b, and Figure 5, which present very often better results from sensors sets (88.3–100%) that from the individual sensors, for any volunteer. There are sometimes opposite cases, but only when the individual sensor (with lower recognition for some activity or some volunteer) is applied to a sensor set. In such a situation, this sensor decreases the recognition for the sensor set and this recognition is lower than for the other individual sensor (with higher recognition).

To sum up, the individual sensors have complementary scopes of competences and their mutual exchange depending on the current situation benefits better results than the usage of a rigidly defined sensor set.

Studying sensors’ performance in recognition of six elementary daily living activities, we confirmed that particular sensors show their optimal recognition accuracy at different movements (Table 3). Consequently, due to the complementary competencies of sensors, combining information from multiple different sensors is expected to give more reliable recognition. Unfortunately, in compound actions, true recognition falls into the border area or actually moves from the area of competence of one sensor to another. This remark was a foundation of the presented concept, design. and prototype of an assisted living system with an adaptive sensor contribution.

Based on the comparison of the accuracy of activity recognition by four different assisted living sensors, we built activity-specific sensor priority lists and proposed a multimodal surveillance system with adaptive sensor’s contribution. The setup we used as a model of a sensorized environment in which multiple sensors of possibly different paradigms and performance cooperate in the surveillance of a human. We assumed that sensors not only differ in reliability depending on the subject’s action but also give consistent or contradictory results. We proved this assumption in experiments showing that adding sensors may decrease the correctness of recognition (Table 3). 

Since the sensor data differ in form and refresh rate, sensor-specific data processing was applied first to provide data in a uniform format before fusion. The sensor-independent format was a list of activities ordered by descending detection probability. Activity data matching and fusion are made on the list level and also allows for continuous adaptation of sensors’ contribution to the final result of the network. This proposal has been inspired by a neuromodulatory mechanism, which, although far more complicated, also leads to modulation of the information flow from the senses to the brain.

Biomimetic modulation of a sensor’s contribution in a multisensory assisted living environment puts forward their advantages according to the subject’s behavior. Being aware of limitations present in any human behavior model, we took selected daily living activities as samples in a continuous space of possible behaviors and tried to represent the actual behavior with a measure of similarity to these primitives [34]. In this paper, we showed that sensors, due to the specificity of their work principle, are somewhat ‘specialized’ in the recognition of particular poses or activities. Consequently, if a compound activity is represented by a set of elementary poses of varying contributions (see Section 7.3), the surveillance system, besides other limitations (see Section 7.2), should optimize the flow of sensor data seamlessly. 

Regarding the related works, the main novelty in this paper is the ongoing adaptation of the sensor set dependent on the subject’s behavior. Since the range of activities is virtually unlimited and the prediction of most probable future action is uncertain, given optimization rules had to be proposed and were implemented as:

- Sensor cost—to balance the sensor usage;
- Penalty factor—to balance between multimodal and single mode-switching system;
- Stability check—to maintain decision on detected activity while modifying sensors’ contribution.

Since human activity is a dynamic process, the contribution of the sensors needs to be considered as time-varying. To this point in the design of the multimodal assisted living system with adaptive sensor’s contribution, we proposed to consider conservative and predictive adaptation. The conservative adaptation assumes the sensor contribution is adapted after the activity recognition and, in case other results were issued by the adapted system, raises the stability issue, which can be solved in several ways (e.g., see 6.2). The predictive adaptation requires the use of a subject’s habits database, which has to be created and trained, but it already contains a personalized factor. Moreover, the prediction of behavior is never 100% accurate, something that needs to be taken into consideration in the design of adaptation rules. 

We used four different sensors with quite good performance in the given experimental setup. However, one should consider more difficult or unstable conditions (e.g., lighting) and simplified sensors (e.g., when the energy consumption will be taken into consideration). The maximum error the system will make in activity recognition is expected as equal to the error of the second sensor. 

Conservative adaptation in the two-sensor mode, (*p* > 1) may give erroneous recognition which (according to Table 3) may be inaccurate by 5.9% of cases (activity 4b, sensors C and E). The stability check in conservative adaptation prevents the system from changing the recognition decision based on an inappropriate change of sensors. The proposed new sensor set is applied in a subsequent sensing step and if the previous activity is maintained and the new settings are appropriate, a more accurate recognition will be issued. 

In predictive adaptation, the unexpected behavior may affect the sensor set adaptation making the new proposed set inappropriate. In this case, again one should consider the case that a less accurate sensor will be proposed, and the overall reliability will decrease. Unlike the conservative case, the subject’s history (represented in Figure 7 as the “habits” database) helps to avoid the adaptation mismatch. However, it is worth noting that we used only a single step prediction (i.e., next most probable activity has been taken as a background for sensors adaptation), and future studies are necessary to potentially extend the prediction range to a tree of *n* future activities.

Our studies presented here were performed with the data recorded from specific sensors (including custom sensor-specific software, Figure 2) in the given test environment described by Smoleń [50]. With different sensors, particular findings (such as Table 3) may differ significantly, but a general rule of building sensor set hierarchies is universal and worth follow-up by other scientists developing multimodal human activity sensing systems. Therefore, we found it reasonable to present the system operation in four case studies than to give a quantitative evaluation of setup-specific activity detection efficiency.

The building of such a prototype system combining wearable and infrastructural sensors is the aim of our next project. Also, the question of initial personalization of recognition and data flow rules needs to be considered again in the context of a working prototype.

## 9. Conclusions

Based on the analysis of the performance of four different assisted living sensors in six elementary reversible activity types of the human, we proposed the analysis rules with adaptive sensor contribution. We applied them to the design of an auto-optimizing multimodal surveillance system and studied its behavior in true-to-life assisted-living scenarios, including compound activities. We pointed out the possible advantages of complementary competences of the sensors and confirmed benefits resulting from their adaptive contribution.

The building of such a prototype system combining wearable and infrastructural sensors is the aim of our next project. Also, the question of initial personalization of recognition and data flow rules need to be considered again in the context of a working prototype.

## Figures and Tables

**Table 1:** Sampling frequency (*Fs*) for sensors B ÷ E.

	B (EMG)	C (Pressure)	D (Accelerometer)	E (Video)
Fs (Hz)	200	100	100	25

**Table 2:** Vectors for classification of the selected motor activities.

Sensor	*L* Vector Length	Vector Structure
B (EMG)	320	[EL1 EL2 EL3 EL4 EP1 EP2 EP3 EP4]
C (pressure)	240	[L1 L2 L3 P1 P2 P3]
D (accelerometer)	120	[X Y Z]
E (video)	320	[B1 B2 B3 B4 B5 B6 B7 B8]
BC	560	[EL1 EL2 EL3 EL4 EP1 EP2 EP3 EP4 L1 L2 L3 P1 P2 P3]
BD	440	[EL1 EL2 EL3 EL4 EP1 EP2 EP3 EP4 X Y Z]
BE	640	[EL1 EL2 EL3 EL4 EP1 EP2 EP3 EP4 B1 B2 B3 B4 B5 B6 B7 B8]
CD	360	[L1 L2 L3 P1 P2 P3 X Y Z]
CE	560	[L1 L2 L3 P1 P2 P3 B1 B2 B3 B4 B5 B6 B7 B8]
DE	440	[X Y Z B1 B2 B3 B4 B5 B6 B7 B8]
BCD	680	[EL1 EL2 EL3 EL4 EP1 EP2 EP3 EP4 L1 L2 L3 P1 P2 P3 X Y Z]
BCE	880	[EL1 EL2 EL3 EL4 EP1 EP2 EP3 EP4 L1 L2 L3 P1 P2 P3 B1 B2 B3 B4 B5 B6 B7 B8]

**Table 3:** Table of recognition *R_s_a_* (in %) of motion activities 1a ÷ 6b in the test set for all volunteers together for sensors B ÷ E and their sets. Calculation errors *U_s_a_* are placed in orange areas.

	1a	1b	2a	2b	3a	3b	4a	4b	5a	5b	6a	6b	ALL
B	96.9	100.0	99.5	98.5	99.0	99.3	99.1	98.6	97.9	98.2	96.0	97.6	98.4
	3.9	0.0	1.5	3.7	3.4	1.8	3.2	3.1	3.7	3.5	12.7	7.5	1.8
C	90.8	91.8	95.7	96.7	94.9	92.4	95.3	93.6	87.0	88.2	94.9	97.1	93.1
	10.0	10.8	12.4	15.5	6.9	6.4	10.2	7.6	15.0	14.6	14.1	8.2	5.7
D	95.2	97.2	95.5	94.2	96.6	95.1	98.4	97.6	97.9	99.3	96.5	96.0	96.7
	17.2	11.2	15.8	15.4	6.6	8.8	3.2	4.1	2.8	1.9	12.3	12.1	5.2
E	99.7	99.5	95.5	95.5	99.3	97.6	96.0	79.8	99.3	99.3	91.7	92.3	95.5
	1.1	1.6	17.7	18.8	1.8	4.4	7.9	25.0	1.7	1.7	9.1	8.4	4.4
BC	99.2	100.0	99.2	99.5	99.3	99.0	98.4	98.1	97.3	97.5	96.0	97.9	98.4
	1.9	0.0	1.9	3.1	2.3	2.0	5.2	5.2	4.5	5.9	12.3	5.7	2.3
BD	98.2	100.0	99.5	99.2	99.5	99.8	99.3	99.5	99.5	100.0	96.3	97.9	99.1
	3.4	0.0	1.5	2.4	2.2	1.1	2.2	1.4	1.4	0.0	12.8	7.0	1.6
BE	99.0	100.0	99.7	100.0	99.5	99.8	100.0	96.5	99.5	99.8	96.3	97.1	99.0
	2.1	0.0	1.1	0.0	1.5	1.1	0.0	11.4	1.4	0.9	11.7	10.1	2.1
CD	96.4	99.2	97.5	98.2	99.3	98.3	97.9	97.9	98.6	99.3	95.2	96.8	97.9
	8.6	2.7	8.3	6.3	1.8	4.8	5.4	5.5	2.5	2.0	14.0	8.5	3.1
CE	99.5	99.7	99.7	100.0	99.5	97.3	98.8	94.1	98.9	99.5	95.2	96.5	98.3
	1.6	1.1	1.1	0.0	1.5	4.9	2.6	14.9	2.8	1.8	12.5	10.5	2.5
DE	99.7	100.0	99.7	100.0	99.8	98.0	99.1	96.2	99.5	99.8	97.6	97.1	98.9
	1.1	0.0	1.1	0.0	1.1	4.0	2.5	11.4	1.4	0.8	5.8	6.4	1.2
BCD	99.0	100.0	99.7	99.7	99.5	99.5	98.6	99.8	99.3	99.5	96.5	97.9	99.1
	2.6	0.0	1.2	1.5	1.5	1.5	4.2	1.0	1.7	1.2	12.0	5.7	1.5
BCE	98.7	100.0	100.0	99.7	99.8	99.3	99.5	97.2	98.9	99.5	96.5	97.9	98.9
	2.8	0.0	0.0	1.1	1.1	1.8	1.2	9.2	2.5	1.8	10.6	5.7	1.6
CDE	99.7	100.0	100.0	100.0	99.5	98.0	99.3	96.0	99.5	99.5	95.7	97.3	98.7
	1.1	0.0	0.0	0.0	1.5	4.6	1.6	14.3	1.3	1.8	11.1	7.3	2.1
BDE	99.2	100.0	100.0	99.7	100.0	99.8	100.0	98.4	100.0	100.0	96.5	97.9	99.3
	1.8	0.0	0.0	1.1	0.0	1.1	0.0	7.1	0.0	0.0	11.7	7.0	1.4
BCDE	99.2	100.0	100.0	99.7	99.5	99.3	99.8	98.1	99.3	99.5	96.5	97.9	99.1
	1.8	0.0	0.0	1.1	1.5	1.8	1.0	7.1	1.7	1.8	10.6	6.1	1.6

**Table 4:** Table of recognition *R_s_V_* (in %) of all motor activities for volunteers V1 ÷ V20 in test set for sensors B ÷ E and their sets. Calculation errors *U_s_V_* are placed in orange areas.

	W1	W2	W3	W4	W5	W6	W7	W8	W9	W10	W11	W12	W13	W14	W15	W16	W17	W18	W19	W20	…	ALL
B	99.1	94.8	98.4	98.3	98.7	99.1	99.6	97.7	98.3	98.0	98.4	99.6	92.6	100.0	99.6	99.1	97.3	99.6	99.6	100.0	...	98.4
	2.0	7.8	2.4	4.6	3.1	1.9	1.5	4.1	3.3	4.5	3.7	1.4	15.8	0.0	1.4	2.9	4.3	1.1	1.1	0.0	...	1.1
C	98.2	94.8	98.0	97.9	92.3	97.4	97.3	94.9	95.7	91.2	94.4	88.0	85.3	97.1	87.3	88.9	75.2	94.7	93.5	98.4	...	93.1
	2.5	5.6	3.3	2.2	11.6	5.9	5.0	7.8	4.1	12.5	7.2	17.2	15.9	3.3	13.4	13.6	22.7	13.1	11.8	4.4	...	3.3
D	96.9	93.1	99.2	96.9	98.3	98.7	100.0	76.2	93.1	97.6	99.2	99.2	91.1	98.2	100.0	98.3	94.6	100.0	100.0	100.0	...	96.7
	3.9	11.1	1.9	6.5	2.5	3.2	0.0	33.8	11.3	4.1	2.7	1.9	17.2	2.9	0.0	2.5	9.1	0.0	0.0	0.0	...	1.5
E	97.4	95.3	95.9	88.8	97.9	99.1	99.6	97.7	94.4	93.6	99.2	81.0	96.5	94.9	99.2	98.3	92.3	97.3	97.7	94.8	...	95.5
	6.4	9.7	7.4	23.4	3.6	1.9	2.0	3.1	10.5	14.7	1.9	31.8	5.3	16.2	1.9	3.9	16.1	4.7	4.5	11.2	...	5.8
BC	99.6	96.6	99.2	99.7	99.6	99.1	99.6	98.6	98.7	96.0	98.8	99.2	91.1	100.0	99.2	100.0	94.1	100.0	100.0	99.6	...	98.4
	1.6	5.9	1.9	1.1	1.5	1.9	1.5	3.3	3.1	8.8	3.6	2.0	15.1	0.0	1.9	0.0	6.8	0.0	0.0	1.4	...	1.1
BD	99.1	95.7	99.2	99.0	100.0	100.0	100.0	98.6	99.1	99.2	99.6	99.6	93.8	100.0	100.0	100.0	99.1	100.0	100.0	100.0	...	99.1
	1.7	8.4	1.9	2.9	0.0	0.0	0.0	2.4	2.9	2.6	1.4	1.4	15.4	0.0	0.0	0.0	3.0	0.0	0.0	0.0	...	1.1
BE	99.1	96.1	99.6	99.7	100.0	99.6	100.0	99.1	99.6	95.6	100.0	100.0	91.9	100.0	100.0	100.0	99.5	99.6	100.0	100.0	...	99.0
	2.0	8.2	1.4	1.1	0.0	1.4	0.0	2.1	1.5	14.4	0.0	0.0	16.4	0.0	0.0	0.0	1.5	1.1	0.0	0.0	...	1.4
CD	99.6	97.0	99.6	99.3	98.7	97.4	99.6	92.1	99.1	97.2	100.0	97.1	93.0	100.0	100.0	100.0	88.3	99.6	99.2	100.0	...	97.9
	1.6	6.9	1.4	1.7	2.3	6.3	1.5	12.5	1.9	6.8	0.0	5.8	15.8	0.0	0.0	0.0	13.3	1.1	1.9	0.0	...	1.2
CE	99.1	97.0	99.6	99.7	100.0	100.0	100.0	99.5	98.7	94.0	98.8	97.1	94.2	98.5	99.6	98.7	90.5	99.6	100.0	100.0	...	98.3
	2.0	6.0	1.4	1.0	0.0	0.0	0.0	1.6	3.1	18.3	3.0	6.2	11.4	4.2	1.4	3.1	16.6	1.1	0.0	0.0	...	2.0
DE	99.1	96.1	99.2	98.6	99.1	100.0	100.0	99.1	98.7	95.6	100.0	99.2	98.8	98.5	99.6	99.6	99.5	100.0	100.0	97.2	...	98.9
	1.8	8.4	1.9	4.1	2.1	0.0	0.0	2.3	2.3	14.4	0.0	2.9	2.1	4.1	1.4	1.5	1.4	0.0	0.0	6.5	...	1.3
BCD	99.6	97.4	99.6	99.7	100.0	99.6	100.0	99.1	98.7	98.0	100.0	100.0	94.6	100.0	100.0	100.0	95.9	100.0	100.0	100.0	...	99.1
	1.6	6.0	1.4	1.1	0.0	1.5	0.0	2.1	3.1	5.3	0.0	0.0	14.2	0.0	0.0	0.0	4.8	0.0	0.0	0.0	...	1.0
BCE	99.6	96.6	99.6	99.3	99.6	100.0	99.6	99.5	98.7	96.0	100.0	99.6	94.6	99.6	100.0	100.0	96.4	100.0	100.0	100.0	...	98.9
	1.5	8.2	1.4	1.5	1.5	0.0	1.5	1.6	3.1	11.8	0.0	1.4	11.4	1.4	0.0	0.0	5.7	0.0	0.0	0.0	...	1.1
CDE	100.0	97.0	99.6	99.7	100.0	100.0	100.0	99.5	99.6	94.0	100.0	97.9	94.6	100.0	100.0	99.6	93.7	99.6	100.0	100.0	...	98.7
	0.0	7.4	1.4	1.0	0.0	0.0	0.0	1.6	1.4	18.3	0.0	5.8	11.4	0.0	0.0	1.5	9.8	1.1	0.0	0.0	...	1.6
BDE	100.0	97.0	99.6	99.7	100.0	100.0	100.0	99.5	99.6	97.2	100.0	100.0	94.6	100.0	100.0	100.0	99.5	100.0	100.0	100.0	...	99.3
	0.0	8.3	1.4	1.3	0.0	0.0	0.0	1.6	1.5	9.2	0.0	0.0	14.3	0.0	0.0	0.0	1.5	0.0	0.0	0.0	...	1.1
BCDE	100.0	96.1	99.6	99.7	100.0	100.0	100.0	99.5	99.1	96.8	100.0	99.6	94.6	100.0	100.0	100.0	96.8	100.0	100.0	100.0	...	99.1
	0.0	9.3	1.4	1.3	0.0	0.0	0.0	1.6	1.9	9.1	0.0	1.4	11.4	0.0	0.0	0.0	3.5	0.0	0.0	0.0	...	1.0

**Table 5:** From sensors (in %) to the compound action recognition, “searching on the shelf”.

Time (s)	Pose	B (EMG)	C (Pressure)	D (Accelerometer)	E (Video)
0	4a	60.0	7.5	7.5	25.0
1.4	4b	42.5	7.5	35.0	17.5
1.9	3a	42.5	5.0	42.5	10.0
3.2	3b	42.5	5.0	42.5	10.0
3.7	5a	17.5	5.0	60.0	17.5
4.9	5b	17.5	5.0	60.0	17.5
5.1	1a	42.5	5.0	42.5	10.0
6.7	1b	60.0	5.0	25.0	10.0

## Appendix A

Matrices of the recognition errors (in %) of the individual motor activities 1a ÷ 6b in the test set for all people together for sets of sensors B ÷ E (BC, BD, BE, CD, CE, DE, BCD, BCE, CDE, BDE, BCDE) are shown in Table A1, Table A2, Table A3, Table A4, Table A5, Table A6, Table A7, Table A8, Table A9, Table A10 and Table A11. The first column 1a ÷ 6b describes the types of activities recognized by the classifier, the first line is the description of the performed activities. The percentage of correct recognition for the individual activities is therefore placed on a diagonal matrix.

**Table A1:** Table of recognition errors (in %) of 12 motor activities 1a ÷ 6b in test set for all volunteers together for sensor BC.

BC		Performed Activity											
		1a	1b	2a	2b	3a	3b	4a	4b	5a	5b	6a	6b
**Recognized Activity**	1a	99.2	0.0	0.0	0.0	0.2	0.0	0.0	0.0	0.0	0.0	0.0	0.0
	1b	0.0	100.0	0.0	0.0	0.0	0.0	0.0	0.0	0.0	0.0	0.0	0.0
	2a	0.5	0.0	99.2	0.5	0.0	0.0	0.0	0.0	0.0	0.0	0.0	0.0
	2b	0.0	0.0	0.3	99.5	0.0	0.0	0.0	0.0	0.0	0.0	0.0	0.0
	3a	0.0	0.0	0.0	0.0	99.3	0.2	0.0	0.0	0.0	0.0	0.0	0.0
	3b	0.0	0.0	0.0	0.0	0.0	99.0	0.0	0.0	0.0	0.0	0.0	0.0
	4a	0.3	0.0	0.0	0.0	0.0	0.2	98.4	1.9	2.1	0.5	0.3	0.0
	4b	0.0	0.0	0.3	0.0	0.0	0.2	1.6	98.1	0.0	2.1	0.0	0.3
	5a	0.0	0.0	0.0	0.0	0.5	0.0	0.0	0.0	97.3	0.0	0.0	0.0
	5b	0.0	0.0	0.3	0.0	0.0	0.2	0.0	0.0	0.7	97.5	0.0	0.0
	6a	0.0	0.0	0.0	0.0	0.0	0.0	0.0	0.0	0.0	0.0	96.0	1.9
	6b	0.0	0.0	0.0	0.0	0.0	0.0	0.0	0.0	0.0	0.0	3.7	97.9

**Table A2:** Matrix of recognition errors (in %) of 12 motor activities 1a ÷ 6b in test set for all volunteers together for sensor BD.

BD		Performed Activity											
		1a	1b	2a	2b	3a	3b	4a	4b	5a	5b	6a	6b
**Recognized Activity**	1a	98.2	0.0	0.0	0.0	0.0	0.0	0.0	0.0	0.0	0.0	0.0	0.0
	1b	0.0	100.0	0.3	0.0	0.0	0.0	0.0	0.0	0.0	0.0	0.0	0.0
	2a	1.0	0.0	99.5	0.8	0.0	0.0	0.0	0.0	0.0	0.0	0.0	0.0
	2b	0.0	0.0	0.0	99.2	0.0	0.0	0.0	0.0	0.2	0.0	0.0	0.0
	3a	0.0	0.0	0.0	0.0	99.5	0.0	0.2	0.0	0.0	0.0	0.0	0.0
	3b	0.0	0.0	0.0	0.0	0.0	99.8	0.0	0.2	0.0	0.0	0.0	0.0
	4a	0.3	0.0	0.0	0.0	0.2	0.0	99.3	0.2	0.2	0.0	0.0	0.0
	4b	0.0	0.0	0.0	0.0	0.2	0.2	0.5	99.5	0.0	0.0	0.3	0.0
	5a	0.5	0.0	0.3	0.0	0.0	0.0	0.0	0.0	99.5	0.0	0.0	0.0
	5b	0.0	0.0	0.0	0.0	0.0	0.0	0.0	0.0	0.0	100.0	0.0	0.0
	6a	0.0	0.0	0.0	0.0	0.0	0.0	0.0	0.0	0.0	0.0	96.3	2.1
	6b	0.0	0.0	0.0	0.0	0.0	0.0	0.0	0.0	0.0	0.0	3.5	97.9

**Table A3:** Matrix of recognition errors (in %) of 12 motor activities 1a ÷ 6b in test set for all volunteers together for sensor BE.

BE		Performed Activity											
		1a	1b	2a	2b	3a	3b	4a	4b	5a	5b	6a	6b
**Recognized Activity**	1a	99.0	0.0	0.0	0.0	0.0	0.0	0.0	0.0	0.0	0.0	0.0	0.0
	1b	0.0	100.0	0.0	0.0	0.0	0.0	0.0	0.0	0.0	0.0	0.0	0.0
	2a	0.5	0.0	99.7	0.0	0.0	0.0	0.0	0.0	0.0	0.0	0.0	0.0
	2b	0.0	0.0	0.0	100.0	0.2	0.0	0.0	0.0	0.0	0.0	0.0	0.0
	3a	0.0	0.0	0.0	0.0	99.5	0.0	0.0	0.0	0.0	0.0	0.0	0.0
	3b	0.0	0.0	0.0	0.0	0.0	99.8	0.0	0.0	0.0	0.0	0.0	0.0
	4a	0.0	0.0	0.0	0.0	0.2	0.2	100.0	3.3	0.5	0.0	0.0	0.0
	4b	0.5	0.0	0.0	0.0	0.0	0.0	0.0	96.5	0.0	0.2	0.0	0.0
	5a	0.0	0.0	0.3	0.0	0.0	0.0	0.0	0.0	99.5	0.0	0.0	0.0
	5b	0.0	0.0	0.0	0.0	0.0	0.0	0.0	0.2	0.0	99.8	0.0	0.0
	6a	0.0	0.0	0.0	0.0	0.0	0.0	0.0	0.0	0.0	0.0	96.3	2.9
	6b	0.0	0.0	0.0	0.0	0.0	0.0	0.0	0.0	0.0	0.0	3.7	97.1

**Table A4:** Matrix of recognition errors (in %) of 12 motor activities 1a ÷ 6b in test set for all volunteers together for sensor CD.

CD		Performed Activity											
		1a	1b	2a	2b	3a	3b	4a	4b	5a	5b	6a	6b
**Recognized Activity**	1a	96.4	0.0	0.0	0.0	0.0	0.0	0.0	0.0	0.2	0.0	0.0	0.0
	1b	0.5	99.2	0.0	0.0	0.0	0.0	0.0	0.0	0.0	0.2	0.0	0.0
	2a	0.3	0.3	97.5	1.5	0.0	0.0	0.0	0.0	0.5	0.0	0.0	0.0
	2b	0.5	0.0	2.0	98.2	0.0	0.0	0.0	0.0	0.0	0.0	0.0	0.0
	3a	0.0	0.0	0.0	0.0	99.3	0.0	0.0	0.0	0.0	0.0	0.0	0.0
	3b	0.0	0.0	0.0	0.0	0.0	98.3	0.0	0.0	0.0	0.0	0.0	0.0
	4a	0.3	0.0	0.3	0.0	0.7	0.0	97.9	2.1	0.5	0.0	0.0	0.0
	4b	0.5	0.3	0.3	0.0	0.0	1.7	2.1	97.9	0.0	0.5	0.0	0.3
	5a	1.3	0.0	0.0	0.3	0.0	0.0	0.0	0.0	98.6	0.0	0.0	0.0
	5b	0.3	0.3	0.0	0.0	0.0	0.0	0.0	0.0	0.2	99.3	0.0	0.0
	6a	0.0	0.0	0.0	0.0	0.0	0.0	0.0	0.0	0.0	0.0	95.2	2.9
	6b	0.0	0.0	0.0	0.0	0.0	0.0	0.0	0.0	0.0	0.0	4.8	96.8

**Table A5:** Matrix of recognition errors (in %) of 12 motor activities 1a ÷ 6b in test set for all volunteers together for sensor CE.

CE		Performed Activity											
		1a	1b	2a	2b	3a	3b	4a	4b	5a	5b	6a	6b
**Recognized Activity**	1a	99.5	0.0	0.0	0.0	0.0	0.0	0.0	0.0	0.2	0.0	0.0	0.0
	1b	0.0	99.7	0.0	0.0	0.0	0.0	0.0	0.0	0.0	0.0	0.0	0.0
	2a	0.0	0.0	99.7	0.0	0.0	0.2	0.0	0.0	0.0	0.0	0.0	0.0
	2b	0.0	0.0	0.0	100.0	0.0	0.0	0.0	0.0	0.0	0.0	0.0	0.0
	3a	0.0	0.0	0.0	0.0	99.5	0.0	0.0	0.0	0.0	0.0	0.0	0.0
	3b	0.0	0.0	0.0	0.0	0.0	97.3	0.0	0.0	0.0	0.0	0.0	0.0
	4a	0.5	0.0	0.0	0.0	0.5	0.5	98.8	5.9	0.5	0.0	0.0	0.0
	4b	0.0	0.3	0.3	0.0	0.0	2.0	1.2	94.1	0.0	0.5	0.0	0.0
	5a	0.0	0.0	0.0	0.0	0.0	0.0	0.0	0.0	98.9	0.0	0.0	0.0
	5b	0.0	0.0	0.0	0.0	0.0	0.0	0.0	0.0	0.5	99.5	0.0	0.0
	6a	0.0	0.0	0.0	0.0	0.0	0.0	0.0	0.0	0.0	0.0	95.2	3.5
	6b	0.0	0.0	0.0	0.0	0.0	0.0	0.0	0.0	0.0	0.0	4.8	96.5

**Table A6:** Matrix of recognition errors (in %) of 12 motor activities 1a ÷ 6b in test set for all volunteers together for sensor DE.

DE		Performed Activity											
		1a	1b	2a	2b	3a	3b	4a	4b	5a	5b	6a	6b
**Recognized Activity**	1a	99.7	0.0	0.0	0.0	0.0	0.0	0.0	0.0	0.2	0.0	0.0	0.0
	1b	0.0	100.0	0.0	0.0	0.0	0.0	0.0	0.0	0.0	0.2	0.0	0.0
	2a	0.0	0.0	99.7	0.0	0.0	0.2	0.0	0.0	0.0	0.0	0.0	0.0
	2b	0.0	0.0	0.0	100.0	0.0	0.0	0.0	0.0	0.0	0.0	0.0	0.0
	3a	0.0	0.0	0.0	0.0	99.8	0.0	0.7	0.0	0.0	0.0	0.0	0.0
	3b	0.0	0.0	0.0	0.0	0.0	98.0	0.0	0.2	0.0	0.0	0.0	0.0
	4a	0.0	0.0	0.0	0.0	0.0	0.0	99.1	3.5	0.2	0.0	0.0	0.0
	4b	0.0	0.0	0.0	0.0	0.2	1.7	0.2	96.2	0.0	0.0	0.0	0.0
	5a	0.3	0.0	0.0	0.0	0.0	0.0	0.0	0.0	99.5	0.0	0.0	0.0
	5b	0.0	0.0	0.3	0.0	0.0	0.0	0.0	0.0	0.0	99.8	0.0	0.0
	6a	0.0	0.0	0.0	0.0	0.0	0.0	0.0	0.0	0.0	0.0	97.6	2.9
	6b	0.0	0.0	0.0	0.0	0.0	0.0	0.0	0.0	0.0	0.0	2.4	97.1

**Table A7:** Matrix of recognition errors (in %) of 12 motor activities 1a ÷ 6b in test set for all volunteers together for sensor BCD.

BCD		Performed Activity											
		1a	1b	2a	2b	3a	3b	4a	4b	5a	5b	6a	6b
**Recognized Activity**	1a	99.0	0.0	0.0	0.0	0.0	0.0	0.0	0.0	0.0	0.0	0.0	0.0
	1b	0.0	100.0	0.0	0.0	0.0	0.0	0.0	0.0	0.0	0.0	0.0	0.0
	2a	0.3	0.0	99.7	0.3	0.0	0.0	0.0	0.0	0.0	0.0	0.0	0.0
	2b	0.0	0.0	0.0	99.7	0.0	0.0	0.0	0.0	0.0	0.0	0.0	0.0
	3a	0.0	0.0	0.0	0.0	99.5	0.0	0.0	0.0	0.0	0.0	0.0	0.0
	3b	0.0	0.0	0.0	0.0	0.0	99.5	0.0	0.0	0.0	0.0	0.0	0.0
	4a	0.3	0.0	0.0	0.0	0.5	0.0	98.6	0.2	0.7	0.0	0.0	0.0
	4b	0.0	0.0	0.3	0.0	0.0	0.5	1.4	99.8	0.0	0.5	0.3	0.3
	5a	0.5	0.0	0.0	0.0	0.0	0.0	0.0	0.0	99.3	0.0	0.0	0.0
	5b	0.0	0.0	0.0	0.0	0.0	0.0	0.0	0.0	0.0	99.5	0.0	0.0
	6a	0.0	0.0	0.0	0.0	0.0	0.0	0.0	0.0	0.0	0.0	96.5	1.9
	6b	0.0	0.0	0.0	0.0	0.0	0.0	0.0	0.0	0.0	0.0	3.2	97.9

**Table A8:** Matrix of recognition errors (in %) of 12 motor activities 1a ÷ 6b in test set for all volunteers together for sensor BCE.

BCE		Performed Activity											
		1a	1b	2a	2b	3a	3b	4a	4b	5a	5b	6a	6b
**Recognized Activity**	1a	98.7	0.0	0.0	0.0	0.0	0.0	0.0	0.0	0.0	0.0	0.0	0.0
	1b	0.0	100.0	0.0	0.0	0.0	0.0	0.0	0.0	0.0	0.0	0.0	0.0
	2a	0.3	0.0	100.0	0.0	0.2	0.0	0.0	0.0	0.0	0.0	0.0	0.0
	2b	0.0	0.0	0.0	99.7	0.0	0.0	0.0	0.0	0.0	0.0	0.0	0.0
	3a	0.0	0.0	0.0	0.0	99.8	0.0	0.0	0.0	0.0	0.0	0.0	0.0
	3b	0.0	0.0	0.0	0.0	0.0	99.3	0.0	0.0	0.0	0.0	0.0	0.0
	4a	0.5	0.0	0.0	0.3	0.0	0.2	99.5	2.8	0.7	0.0	0.0	0.0
	4b	0.3	0.0	0.0	0.0	0.0	0.5	0.5	97.2	0.2	0.5	0.0	0.0
	5a	0.3	0.0	0.0	0.0	0.0	0.0	0.0	0.0	98.9	0.0	0.0	0.0
	5b	0.0	0.0	0.0	0.0	0.0	0.0	0.0	0.0	0.2	99.5	0.0	0.0
	6a	0.0	0.0	0.0	0.0	0.0	0.0	0.0	0.0	0.0	0.0	96.5	2.1
	6b	0.0	0.0	0.0	0.0	0.0	0.0	0.0	0.0	0.0	0.0	3.5	97.9

**Table A9:** Matrix of recognition errors (in %) of 12 motor activities 1a ÷ 6b in test set for all volunteers together for sensor CDE.

CDE		Performed Activity											
		1a	1b	2a	2b	3a	3b	4a	4b	5a	5b	6a	6b
**Recognized activity**	1a	99.7	0.0	0.0	0.0	0.0	0.0	0.0	0.0	0.0	0.0	0.0	0.0
	1b	0.0	100.0	0.0	0.0	0.0	0.0	0.0	0.0	0.0	0.0	0.0	0.0
	2a	0.3	0.0	100.0	0.0	0.0	0.0	0.0	0.0	0.0	0.0	0.0	0.0
	2b	0.0	0.0	0.0	100.0	0.0	0.0	0.0	0.0	0.0	0.0	0.0	0.0
	3a	0.0	0.0	0.0	0.0	99.5	0.0	0.0	0.0	0.0	0.0	0.0	0.0
	3b	0.0	0.0	0.0	0.0	0.0	98.0	0.0	0.0	0.0	0.0	0.0	0.0
	4a	0.0	0.0	0.0	0.0	0.5	0.2	99.3	4.0	0.2	0.0	0.0	0.0
	4b	0.0	0.0	0.0	0.0	0.0	1.7	0.7	96.0	0.0	0.5	0.0	0.0
	5a	0.0	0.0	0.0	0.0	0.0	0.0	0.0	0.0	99.5	0.0	0.0	0.0
	5b	0.0	0.0	0.0	0.0	0.0	0.0	0.0	0.0	0.2	99.5	0.0	0.0
	6a	0.0	0.0	0.0	0.0	0.0	0.0	0.0	0.0	0.0	0.0	95.7	2.7
	6b	0.0	0.0	0.0	0.0	0.0	0.0	0.0	0.0	0.0	0.0	4.3	97.3

**Table A10:** Matrix of recognition errors (in %) of 12 motor activities 1a ÷ 6b in test set for all volunteers together for sensor BDE.

BDE		Performed Activity											
		1a	1b	2a	2b	3a	3b	4a	4b	5a	5b	6a	6b
**Recognized Activity**	1a	99.2	0.0	0.0	0.0	0.0	0.0	0.0	0.0	0.0	0.0	0.0	0.0
	1b	0.0	100.0	0.0	0.0	0.0	0.0	0.0	0.0	0.0	0.0	0.0	0.0
	2a	0.3	0.0	100.0	0.0	0.0	0.0	0.0	0.0	0.0	0.0	0.0	0.0
	2b	0.0	0.0	0.0	99.7	0.0	0.0	0.0	0.0	0.0	0.0	0.0	0.0
	3a	0.0	0.0	0.0	0.0	100.0	0.0	0.0	0.0	0.0	0.0	0.0	0.0
	3b	0.0	0.0	0.0	0.0	0.0	99.8	0.0	0.0	0.0	0.0	0.0	0.0
	4a	0.3	0.0	0.0	0.3	0.0	0.0	100.0	1.6	0.0	0.0	0.0	0.0
	4b	0.3	0.0	0.0	0.0	0.0	0.2	0.0	98.4	0.0	0.0	0.0	0.0
	5a	0.0	0.0	0.0	0.0	0.0	0.0	0.0	0.0	100.0	0.0	0.0	0.0
	5b	0.0	0.0	0.0	0.0	0.0	0.0	0.0	0.0	0.0	100.0	0.0	0.0
	6a	0.0	0.0	0.0	0.0	0.0	0.0	0.0	0.0	0.0	0.0	96.5	2.1
	6b	0.0	0.0	0.0	0.0	0.0	0.0	0.0	0.0	0.0	0.0	3.5	97.9

**Table A11:** Matrix of recognition errors (in %) of 12 motor activities 1a ÷ 6b in test set for all volunteers together for sensor BCDE.

BCDE		Performed Activity											
		1a	1b	2a	2b	3a	3b	4a	4b	5a	5b	6a	6b
**Recognized Activity**	1a	99.2	0.0	0.0	0.0	0.0	0.0	0.0	0.0	0.0	0.0	0.0	0.0
	1b	0.0	100.0	0.0	0.0	0.0	0.0	0.0	0.0	0.0	0.0	0.0	0.0
	2a	0.0	0.0	100.0	0.0	0.0	0.0	0.0	0.0	0.2	0.0	0.0	0.0
	2b	0.0	0.0	0.0	99.7	0.0	0.0	0.0	0.0	0.0	0.0	0.0	0.0
	3a	0.0	0.0	0.0	0.0	99.5	0.0	0.0	0.0	0.0	0.0	0.0	0.0
	3b	0.0	0.0	0.0	0.0	0.0	99.3	0.0	0.0	0.0	0.0	0.0	0.0
	4a	0.3	0.0	0.0	0.3	0.5	0.2	99.8	1.9	0.5	0.0	0.0	0.0
	4b	0.3	0.0	0.0	0.0	0.0	0.5	0.2	98.1	0.0	0.5	0.0	0.0
	5a	0.3	0.0	0.0	0.0	0.0	0.0	0.0	0.0	99.3	0.0	0.0	0.0
	5b	0.0	0.0	0.0	0.0	0.0	0.0	0.0	0.0	0.0	99.5	0.0	0.0
	6a	0.0	0.0	0.0	0.0	0.0	0.0	0.0	0.0	0.0	0.0	96.5	2.1
	6b	0.0	0.0	0.0	0.0	0.0	0.0	0.0	0.0	0.0	0.0	3.5	97.9
